# Assessing Effectiveness and Costs in Robot-Mediated Lower Limbs Rehabilitation: A Meta-Analysis and State of the Art

**DOI:** 10.1155/2018/7492024

**Published:** 2018-06-04

**Authors:** Giorgio Carpino, Alessandra Pezzola, Michele Urbano, Eugenio Guglielmelli

**Affiliations:** ^1^Laboratory of Biomedical Robotics and Biomicrosystems, Università Campus Bio-Medico di Roma, 00128 Rome, Italy; ^2^Polyclinic General Direction, Università Campus Bio-Medico di Roma, 00128 Rome, Italy

## Abstract

Robots were introduced in rehabilitation in the 90s to meet different needs, that is, reducing the physical effort of therapists. This work consists of a meta-analysis of robot-mediated lower limbs rehabilitation for stroke-affected patients; it aims at evaluating the effectiveness of the robotic approach through the use of wearable robots or operational machines with respect to the conventional approach (i.e., manual rehabilitation therapy). The primary assessed outcome is the patient's ability to recover walking independence, whereas the secondary outcome is the average walking speed. The therapy acceptability and the treatment costs are also assessed. The assessment shows that the robot-mediated therapy is more effective than the conventional one in reaching the primary outcome. As for the secondary outcome, there is no significant difference between the robotic (wearable robots or operational machines) and the conventional approach. Rehabilitation using wearable robots has a greater acceptability than the conventional one. This does not apply to operational machines. The cost of robotic treatment with wearable robots ranges from double to triple the cost of the conventional approach. On the contrary, rehabilitation using operational machines costs the same as the conventional treatment. Robotic rehabilitation based on operational machines is the most cost-effective approach.

## 1. Introduction

The introduction of robots in rehabilitation therapy dates back to the 1990s [1]. Since then, the rehabilitation therapy has been led by the following factors: (i) modern medicine is based on objective assessments and quantitative benchmarking of the impact of different therapeutic approaches; (ii) conventional rehabilitation therapy (i.e., manual rehabilitation therapy) is an intense, time-consuming activity, which requires high physical effort for health workers; (iii) recent studies on neuroplasticity related to functional recovery in patients with brain injuries have highlighted that patients benefit from activity-dependent rehabilitation therapies. These factors usually require the execution of repetitive exercises, aimed at a well-defined goal. The patient has to have an active role during the rehabilitation session in order to stimulate the whole system of sensorimotor coordination, including the stages of imagination and planning of the motor task. Therefore, there are unmet or not completely met needs in the conventional approach to the motor rehabilitation such as availability of measurable outcomes, repeatability of the rehabilitation tasks, and active patient engagement.

Robotic solutions for assisted therapy meet all these needs [2, 3]. Rehabilitation robotics is a new technological branch related to the application of robot technology in medical fields. Nowadays, rehabilitation robots are a key enabling technologies to help people who suffer limb movement disorder to restore the normal physiological muscular activity with the possibility of gain-measurable outcomes. Robot-mediated rehabilitation aims at developing new solutions for assisted therapy, thus allowing an objective functional assessment of patients. Due to the advantages of their accuracy and reliability, rehabilitation robots can provide an effective way to improve the outcome of stroke or postsurgical rehabilitation. Robotic technology offers (i) accuracy, precision, and simple tools for the modelling of the human behaviour; (ii) repeatable and continuous movements of the human districts to rehabilitate; and (iii) active engagement of the patients during the rehabilitation tasks, that is, through virtual reality-based exercises.

Robotic devices for rehabilitation fall into two broad categories (i.e., the ones considered in this paper) based on the relationship between the movements of the human body and those of the machine [4]:

- Operational machines: The physical interface between the robot and human body is in a defined part of the body, usually the effectors. For these machines, the trajectories of the robot end-effector and the human end-effector in the operational space are physically coupled. In the joint space, instead, the trajectories of the robot joints and the human joints can be significantly different, so that, kinematic schemes can also be selected based on only the specific requirements of the target application scenario.
- Wearable robots: In these machines, a large portion of the human body (typically the whole affected limb) is in continuous physical contact with the robot. In most cases, a biomimetic exoskeleton kinematic structure is selected. Therefore, not only the trajectories of the robot end-effector and the human end-effector are the same in the operational space but also the trajectories of the robot joints approximate those of the human joints in the joint space. These systems require advanced biomechatronic design approaches in order to mimic human-like joints motion, while minimizing invasiveness for the patient in terms of weight, dimensions, and so on. To overcome these challenging problems, nonbiomimetic wearable robots are also currently under investigation in a few pilot research projects recently launched in Europe and the U.S. [5, 6].

Wearable robots for walking rehabilitation can be divided in turn into nonportable and portable systems, depending on whether they are fixed to a specific environment or not. Portable systems are autonomous while nonportable systems require a source of energy. Rehabilitation wearable robots can also be classified as robots for rehabilitation on treadmill and robots for overground walking rehabilitation [7, 8].

Lokomat (Hocoma AG, Volketswil, Switzerland), trade name for the DGO (Driven Gait Orthosis), is one of the most widely used wearable robots. It is a nonportable robot for treadmill rehabilitation, with an anthropomorphic nonredundant structure. It assists and guides the hip and knee movements in the sagittal plane, while the ankle joint is not actuated [9]. LOPES (lower-extremity powered exoskeleton) is another example of nonportable robot. It assists flexion/extension and abduction/adduction of the hip and flexion/extension of the knee [10]. ALEX (active leg exoskeleton) is a wearable robot for treadmill rehabilitation with two actuated degrees of freedom, which allow movement in the sagittal plane of the hip and knee joints [11]. AutoAmbulator and Walkbot are nonportable robotic systems with a mechanical structure very similar to Lokomat [12, 13].

ReWalk (ReWalk Robotics, Marlborough, US) is a mobile exoskeleton that allows overground rehabilitation. The battery and the controllers are inside the backpack that the patient wears on his shoulders. It is primarily used for SCI patients, and it must be used in conjunction with crutches [14].

Gait Trainer GT I (Reha-Stim, Berlin, Germany) is one of the most widespread operational machines for walking rehabilitation, and it consists of two footplates connected with the patient's feet mimicking the walking cycle [15]. Gait Master 4 is another operational machine based on the movement of two footplates moved by a connecting rod-crank system. The footplates allow the movement of the human effector back and forth (to simulate the walk) or up and down (up/down the stairs) [16].

In this paper, the authors provide a cost-effectiveness assessment to compare, in terms of efficiency, conventional rehabilitation therapies to wearable robot-/operational machine-mediated rehabilitation for the treatment of stroke-affected patients. The present work aims at assessing the effectiveness of the two therapeutic approaches and evaluating the costs for both types of procedure.

## 2. Materials and Methods

### 2.1. Assessment of Effectiveness

The authors have carried out a review of the articles at the state of the art, which compares the effectiveness of robot-mediated and conventional rehabilitation in stroke-affected patients. The analysis is based on average recovery of patients treated with the two approaches. The study includes all the articles in the Cochrane review [17] and more recent studies [13, 18–21]. The studies included in this review focus on patients who suffered a first stroke, who are over 18 years old (with an average age ranging between 48 and 71 years old), who have sufficient cognitive and communication skills that allow a correct understanding of the rehabilitation session, and who do not suffer cardiac, psychological, and orthopaedic contraindications. The authors have included studies on patients in acute and subacute phases (time elapsed since the ictal event not exceeding three months), on chronic patients, and on patients with different levels of disability. The patient groups range from patients who are able to walk independently even before the beginning of the rehabilitation therapy to patients who are completely dependent to walk.

All clinical trials are based on the random division of patients into two groups: group A and group B.

- Group A: patients in this group underwent a rehabilitation programme consisting of several sessions of robot-mediated therapy and some additional manual therapy session.
- Group B: patients in this group underwent conventional therapy only.

Depending on the particular study, machines used for the robot-mediated rehabilitation of patients in the group A are wearable robots (Lokomat in most of the articles, but also AutoAmbulator, Anklebot, and Walkbot in one study each) or operational machines (Gait Trainer I in all cases except one where Gate Master 4 is used). In both cases, training with a BWS (Body Weight Support) is carried out; a 30–40% of body weight support during the first rehabilitation session is applied. The body weight support is progressively reduced as the patient recovered his/her locomotor ability.

At the end of the rehabilitation process, the primary outcome is the ability of the patients to recover walking independence. The secondary outcome is the average walking speed. The acceptability of the therapy is assessed taking into account the number of dropouts, that is to say, the number of patients who do not complete the entire rehabilitation process.

The authors have applied the same statistical methods as those used in the Cochrane review [17], although not explicitly described here, but suggested by the homonymous statistical group in the “Cochrane Handbook for Systematic Reviews of Interventions” [22]. The odds ratio (OR) is calculated for each article. It measures the number of patients who recovered walking independence at the end of the robotic treatment compared to the conventional one, as well as to quantify the number of dropouts. The OR is calculated as follows:

(1) $$\begin{array}{c} {\text{OR}}_{i}=\frac{a_{i}\cdot d_{i}}{b_{i}\cdot c_{i}}, \end{array}$$

where *a*_*i*_ is the number of patients in group A who recovered independence in walk at the end of the planned rehabilitation process, *b*_*i*_ is the number of patients in group A who did not recover independence in walk, *c*_*i*_ is the number of patients in group B who recovered independence in walk, and *d*_*i*_ is the number of patients in group B who did not recover independence in walk. Similarly, *a*_*i*_, *b*_*i*_, *c*_*i*_, and *d*_*i*_ can be used to measure the number of patients who completed or not the entire rehabilitation process.

The Mantel–Haenzsel (MH) random-effects method is used for meta-analysis and to calculate the Overall OR, which takes into account the results of each article:

(2) $$\begin{array}{c} {\text{OR}}_{\text{MH}}=\frac{\sum w_{i}^{\text{MH}}\cdot {\text{OR}}_{i}}{\sum w_{i}^{\text{MH}}}, \end{array}$$

with *w*_*i*_^MH^=*b*_*i*_ · *c*_*i*_/*n*_*i*_, where *n*_*i*_=*a*_*i*_+*b*_*i*_+*c*_*i*_+*d*_*i*_. If no participant or if every participant in the study achieved the ability to walk independently, the OR of the *i*th article is considered “not estimable,” and it is, thus, not included in the meta-analysis. When this situation just occurs only in one of the two groups (A or B), a modified formula is used. OR_*i*_ is calculated by adding a factor equal to 0.5 to each element in (1).

The confidence interval (CI) is calculated taking into account the overall standard error SE(ln OR_MH_), according to the following formula:

(3) $$\begin{array}{c} 95\%\text{ CI}=\left[e^{\ln{\text{ OR}}_{\text{MH}}-1,96\cdot \text{SE}\left(\ln{\text{ OR}}_{\text{MH}}\right)},\;e^{\ln{\text{ OR}}_{\text{MH}}+1,96\cdot \text{SE}\left(\ln{\text{ OR}}_{\text{MH}}\right)}\right], \end{array}$$

with $\text{SE}\left(\ln{\text{ OR}}_{\text{MH}}\right)=\sqrt{\left(1/2\right)\left(\left(E/R^{2}\right)+\left(F+G/R\cdot S\right)+\left(H/S^{2}\right)\right)}$, where *R*=∑*a*_*i*_ · *d*_*i*_/*n*_*i*_, *S*=∑*b*_*i*_ · *c*_*i*_/*n*_*i*_, *E*=∑(*a*_*i*_+*d*_*i*_) · *a*_*i*_ · *d*_*i*_/*n*_*i*_^2^, *F*=∑(*a*_*i*_+*d*_*i*_) · *b*_*i*_ · *c*_*i*_/*n*_*i*_^2^, *G*=∑(*b*_*i*_+*c*_*i*_) · *a*_*i*_ · *d*_*i*_/*n*_*i*_^2^, and *H*=∑(*b*_*i*_+*c*_*i*_) · *b*_*i*_ · *c*_*i*_/*n*_*i*_^2^.

If 95%  CI includes value 1 (no effect), the result described by the corresponding OR does not demonstrate a clear effectiveness, and it is not considered significant.

The mean difference (MD) is used to calculate the secondary outcome:

(4) $$\begin{array}{c} {\left(\text{MD}\vec{v}\right)}_{i}={\vec{v}}_{i}^{\text{RT}}-{\vec{v}}_{i}^{\text{CT}}\left(\text{m}/\text{s}\right)\text{,} \end{array}$$

where ${\vec{v}}_{i}^{\text{RT}}$ is the average speed that patients reached at the end of the robotic therapy, and ${\vec{v}}_{i}^{\text{CT}}$ is the average speed that patients reached at the end of the conventional therapy.

The inverse variance (IV) random-effects method is used to calculate the Overall MD:

(5) $$\begin{array}{c} {\left(\text{MD}\vec{v}\right)}_{\text{IV}}=\frac{\sum w_{i}\cdot {\left(\text{MD}\vec{v}\right)}_{i}}{\sum w_{i}}\left(\text{m}/\text{s}\right), \end{array}$$

with $w_{i}=1/\text{SE}{\left(\text{MD}\vec{v}\right)}_{i}^{2}$, where $\text{SE}{\left(\text{MD}\vec{v}\right)}_{i}^{2}$ is the variance of the *i*th article.

The CI is calculated taking into account the overall standard error $\text{SE}{\left(\text{MD}\vec{v}\right)}_{\text{IV}}$, according to the following formula:

(6) $$\begin{array}{c} 95\text{\% 2CI}=\left[{\left(\text{MD}\vec{v}\right)}_{\text{IV}}-1.96\cdot \text{SE}{\left(\text{MD}\vec{v}\right)}_{\text{IV}};{\left(\text{MD}\vec{v}\right)}_{\text{IV}}+1.96\cdot \text{SE}{\left(\text{MD}\vec{v}\right)}_{\text{IV}}\right], \end{array}$$

with $\text{SE}{\left(\text{MD}\vec{v}\right)}_{\text{IV}}=1/\sqrt{\sum^{}w_{i}}$. If 95%  CI includes the value 0 (no effect), the result described by the corresponding MD does not demonstrate a clear effectiveness, and it is not considered significant.

Establishing the abovementioned statistical methods, the authors verified to be able to exactly reproduce the same results reported in the Cochrane review [17] (with a difference of maximum 0.02). Therefore, they carried out the calculations again, considering also the 5 more recent studies [13, 18–21], that perfectly fit in terms of inclusion criteria for the patients and the type of trials with the papers included in the Cochrane review.

### 2.2. Costs Analysis

Although in most of the studies patients were hospitalised for rehabilitation therapy, the authors take into account only the costs of the rehabilitation therapy, whereas hospitalisation costs are not considered, as they are effectively the same in the robotic and conventional approaches. Costs of medications, meals, and electricity are identical because the duration of the rehabilitation process, in all studies included in this review, is the same for patients of group A and B. The same applies to health cost reimbursements from the Lazio Region, which are the same in Italy for the conventional- and robot-mediated therapies [23]:

- *C*_h_^therapist^(€/*h*), hourly cost of a single physiotherapist
- *n*^therapists^, average number of therapists per session per patient
- *t*^session^(min), average session duration
- *n*^sessions^, average number of sessions for the entire rehabilitation process for patients mentioned in the articles.

The hourly cost of conventional therapy (*C*_h_^Conventional^), cost per session (*C*_session_^Conventional^), and cost of the entire rehabilitation process (*C*_total_^Conventional^) are calculated. The cost estimate of the robot-mediated treatment takes into account the abovementioned parameters and also the cost to purchase the robot (*C*_Robot–purchase_ (€)), the number of years to amortise the robot (*y*^amortization^ (years)), and the annual routine maintenance cost (*C*_y_^maintenance^ (€/year)).

The hours of potential use of the robot in a health facility are estimated to calculate the hourly cost of the robot (and the cost per session). Physiotherapists' working shifts are taken into account to get an idea of how many hours per day the robot can be used for rehabilitation sessions. Two possible working shifts were identified in collaboration with the Polyclinic General Direction of Campus Bio-medico (Rome, Italy), amounting to a total of 36 weekly working hours. The cost of the robotic therapy is, therefore, calculated for both shifts:

- “1st case”: robot used 7.12 hours per day, 5 days a week. This is the total amount of weekly working hours of a single therapist.
- “2nd case”: robot used 12 hours per day, 6 days a week. This is the total amount of weekly working hours of two therapists, one working in the morning and the other in the afternoon.

This allows to calculate the hourly cost (*C*_h_^Robot^), the cost per session (*C*_session_^Robot^), and the cost of the entire rehabilitation process (*C*_total_^Robot^) for the robotic therapy.

The cost estimate for the entire training process of the two groups (A and B) takes into account, for both approaches (robotic and conventional), the additional cost of conventional therapy in the clinical trials as extra training for patients in both groups:

(7) $$\begin{array}{c} C_{\text{total}}^{\text{patient}}=C_{\text{total}}^{\left(\text{Robot or Conventional}\right)}+C_{\text{additional}}. \end{array}$$

This means that (7) is calculated taking into account the cost of the specific rehabilitation process (*C*_total_^Robot^ for group A and *C*_total_^Conventional^ for group B), plus the additional cost of conventional therapy extra sessions.

### 2.3. Cost-Effectiveness Analysis

The ICER (incremental cost-effectiveness ratio) is calculated to compare the efficiency of the two approaches (conventional versus robotic) and to determine which one is most cost-effective:

(8) $$\begin{array}{c} \text{ICER}=\frac{C_{A}-C_{B}}{E_{A}-E_{B}}, \end{array}$$

where *C*_A_ and *C*_B_ are the cost of the entire rehabilitation process for robot-mediated therapy and conventional therapy, respectively, whereas *E*_A_ and *E*_B_ measure the effectiveness of each therapy in terms of primary outcome, that is to say, the Overall OR for walking independence, or in terms of secondary outcome, the Overall MD of walking speed.

When calculating the ICER, it is important to distinguish whether the difference in effectiveness is expressed in terms of OR or MD. When the difference in effectiveness (*E*_A_ − *E*_B_) is expressed in terms of Overall OR, *C*_A_ and *C*_B_ are calculated by multiplying the cost of the entire rehabilitation process per patient (*C*_total_^patient^) per the average number of treated patients. When the difference in effectiveness (*E*_A_ − *E*_B_) is expressed in terms of Overall MD, *C*_A_ and *C*_B_ costs are equal to *C*_total_^patient^. In both cases, robot-mediated therapy is more effective than the conventional therapy when the ICER value is low. A low value at the ICER ratio numerator indicates a small difference in cost between the two approaches, whereas a high value at the denominator marks a high OR or MD, which means that the robotic therapy is much more effective than the conventional therapy. The more the OR is >1 or MD is >0, the more the robot-mediated therapy is effective compared to the conventional one. Therefore, the ICER is the difference in terms of cost for the two approaches, weighted by the difference of effectiveness.

## 3. Results and Discussion

### 3.1. Effectiveness

A number of 26 trials are included with a total of 1064 patients, all of them affected by a stroke for the first time. 60% of the patients are men and 40% are women, 70% are affected by ischemic stroke and 30% by haemorrhagic stroke, and 50% have a left hemiparesis and 50% a right hemiparesis. The total number of sessions and their duration and frequency are the same in group A and group B.

The authors have selected the appropriate number of articles to assess the primary and secondary outcomes, as well as the therapy acceptability in three different cases:

1. Robot-mediated therapy for group A based on the use of both wearable robots and operational machines.
2. Robot-mediated therapy for group A based on the use of wearable robots,
3. Robot-mediated therapy for group A based on the use of operational machines.

**Figure 1:**
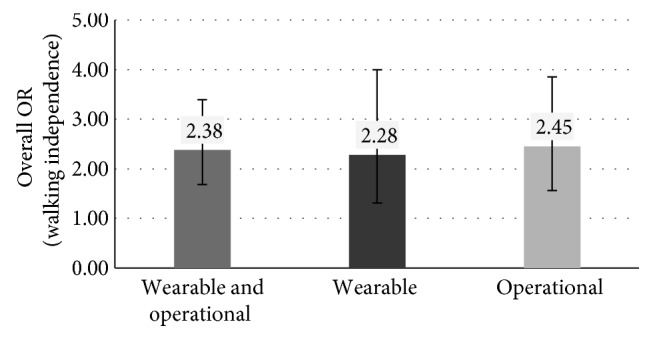
Overall OR (and 95% CI) for independence in walk in the three different cases based on the type of robot used for the rehabilitation of patients in group A.

In all the three cases, the robot-mediated therapy is more effective than the conventional one to recover the patient walking independence (Overall OR > 1) with statistically significant results (*p* value < 0.05 and 95% CI not including value 1), as shown in Figure 1. The OR is equal to 2.38 in the first case (*p* value < 0.0001, 95% CI between 1.68 and 3.39), 2.28 in the second case (*p* value = 0.0038, CI between 1.31 and 4.00), and 2.45 in the third case (*p* value = 0.0001, 95% CI between 1.56 and 3.85).

**Figure 2:**
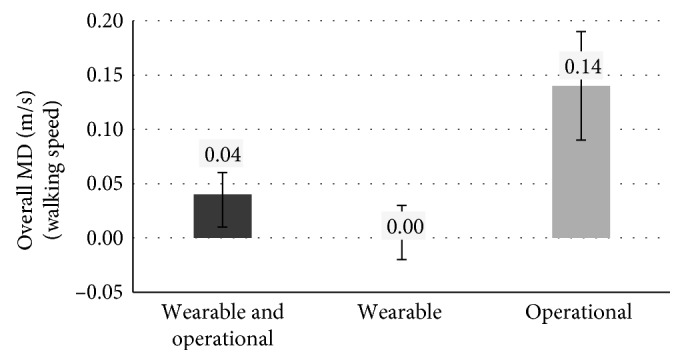
Overall MD (and 95% CI) for walking speed in the three different cases based on the type of robot used for the rehabilitation of patients in group A.

As for the average walking speed achieved at the end of the rehabilitation process, the robotic therapy is slightly more effective than the conventional one (Overall MD is 0.04 m/s, *p* value = 0.0026, 95% CI between 0.01 and 0.06). Operational machines, in particular, are more effective than wearable robots, as shown in Figure 2. Operational machines MD is 0.14 m/s (*p* value < 0.0001, 95% CI between 0.09 and 0.19). Wearable robots have a null MD, which means that wearable robots are as effective as conventional therapy. However, this result is not statistically significant (*p* value = 0.89, 95% CI between −0.02 and 0.03).

**Figure 3:**
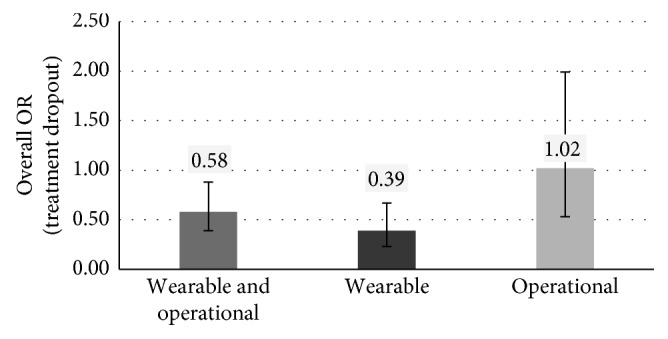
Overall OR (and 95% CI) for dropouts in the three different cases based on the type of robot used for the rehabilitation of patients in group A.

Regarding the acceptability, the robotic therapy is more effective than the conventional one with OR equal to 0.58 (*p* value = 0.01, 95% CI between 0.39 and 0.88), as shown in Figure 3. OR < 1 indicates fewer dropouts. However, the results show differences based on the type of robot used for the rehabilitation of patients in group A. The therapy based on the use of wearable robots is much more acceptable than the conventional one (OR = 0.39, *p* value = 0.0007, 95% CI between 0.23 and 0.67). The therapy based on the use of operational machines has the same acceptability as the conventional one (OR = 1.02). However, this result is not statistically significant (*p* value = 0.95, 95% CI between 0.53 and 1.99).

The authors distinguish between the results for the primary and secondary outcomes depending on patient conditions, that is to say, patients in the subacute or chronic phase.

**Figure 4:**
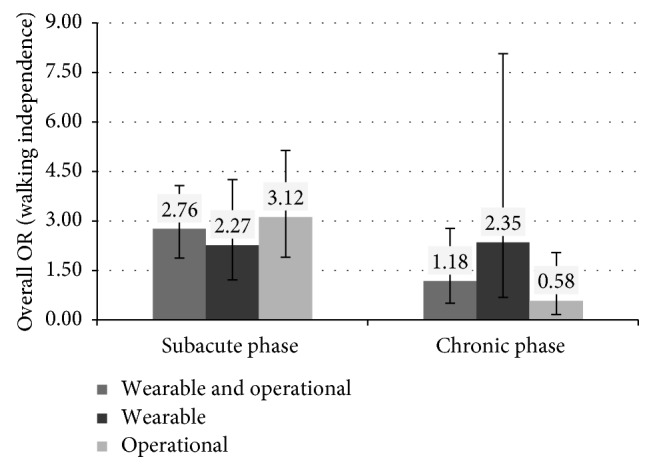
Overall OR (and 95% CI) for walking independence based on the type of robot and the condition of patients (chronic versus subacute phase).

The use of different types of robots to recover the independence in walk produces different results, as shown in Figure 4. The therapy based on the use of wearable robots is more effective than the conventional one for patients both in the chronic (OR = 2.35, *p* value = 0.17, 95% CI between 0.68 and 8.07) and subacute phases (OR = 2.27, *p* value = 0.01, 95% CI between 1.21 and 4.25). The therapy based on the use of operational machines is extremely more effective than the conventional one for patients in the subacute phase (OR = 3.12, *p* < 0.0001, 95% CI between 1.90 and 5.14), but it is less effective than the conventional therapy for chronic patients. However, this result is not statistically significant (OR = 0.58, *p* value = 0.40, 95% CI between 0.16 and 2.04).

**Figure 5:**
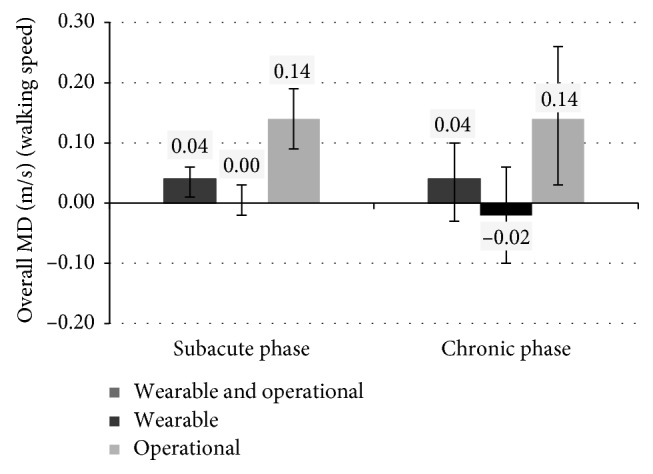
Overall MD (and 95% CI) for walking speed based on the type of robot and the condition of patients (chronic versus subacute phase).

With regards to walking speed, results show no big difference between patients in the subacute or chronic phases, as shown in Figure 5. The therapy based on the use of operational is more effective than the conventional one for both patients in the subacute and chronic phases. For patients in the chronic phase, MD is equal to 0.14 m/s (*p* value = 0.01, 95% CI between 0.03 and 0.26). Similar results are obtained for patients in the subacute phase: MD is equal to 0.14 m/s (*p* value < 0.0001, 95% CI between 0.09 and 0.19). The therapy based on the use of wearable robots is as effective as the conventional one for patients in the subacute phase (MD = 0 m/s), but the result is not statistically significant (*p* value = 0.77, 95% CI between −0.02 and 0.03) and less effective than the conventional one for chronic patients (MD = −0.02 m/s), but the result is still not statistically significant (*p* value = 0.68, 95% CI between −0.10 and 0.06).

### 3.2. Costs

Similar to the effectiveness analysis, three types of comparisons between training costs for the two groups (A and B) are carried out. The first comparison is based on all the articles included in the review, irrespective of the type of robot used for the therapy (operational machine or wearable robot). The other two comparisons focus on two subcases: one based only on studies on wearable robots and the other only on studies on operational machines.

The results are based on a 20 €/h cost per therapist (as per 2014 Collective Bargaining Agreement in Italy) and 5 years to amortise the robot, as per “high-tech medical equipment” tax rate [24]. The annual robot maintenance cost is calculated as 10% of the robot value. For wearable robots, the reference value is set to the Lokomat cost, that is, € 330,000.00. For operational machines, the reference value is set to the Gait Trainer GT I, that is, € 30,000.00. For the robotic therapy in general—regardless of the type of robot—the robot purchase cost is, thus, set equal to € 225,000.00, which is the average between the costs of Lokomat and Gait Trainer GT I weighted by the number of reference articles.

Table 1 shows the parameters used to calculate costs and the relative results, when comparing the conventional therapy to the robot-mediated one, in both the cases described in section “Cost Analysis.” As it could be imagined, the robot-mediated therapy is more expensive and, therefore, less economically sustainable than the conventional one. The cost estimate shows very different results for the therapy based on the use of wearable robot and the one based on operational machines.

Table 2 summarises the hourly cost and total cost (for the full rehabilitation of a patient) for conventional therapy, wearable robots therapy, and operational machines therapy. The therapy with wearable robots costs about three times more than the conventional one considering 12.7 hours of possible use of the robot for 5 days a week (“1st case”). The cost decreases and is about two times more than the cost of the conventional therapy when it is based on 12 hours of possible use of the robot for 6 days a week (“2nd case”). The cost of the therapy with operational machines, on the contrary, is the same as the cost of the conventional therapy in the “1st case” and even lower than the cost of the conventional therapy in the “2nd case.”

### 3.3. Cost-Effectiveness (ICER)

ICER estimates are carried out for the three cases described in section “Effectiveness.” For each case, two different values of ICER are calculated–the first is based on the cost estimate of the robotic therapy in the “1st case” and the second in the “2nd case.”

**Figure 6:**
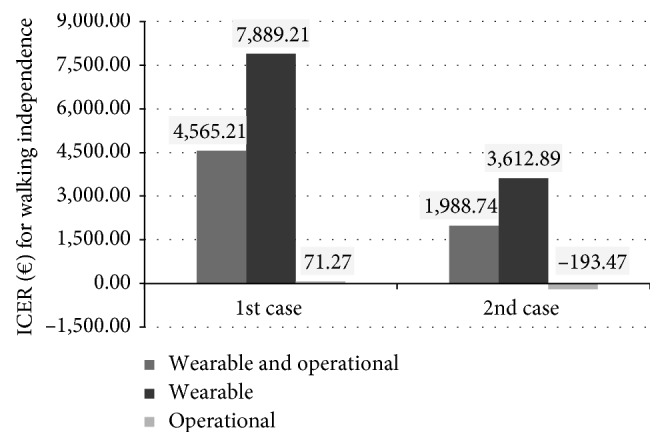
ICER values related to the independence in walk in the different cases based on the type of robot used and on the hours of potential use of the robot.

Figure 6 shows ICER values regarding patients recovering walking independence. For the robot-mediated therapy in general, without considering the type of robot, ICER is € 4,565.21 in the “1st case” and is lower in the “2nd case,” € 1,988.74. Taking into account, instead the type of robot, results are very different. The wearable robots therapy has a higher ICER: € 7,889.21 in the “1st case” and € 3,612.89 in the “2nd case.” The ICER for operational machines therapy is much lower and amounts to € 71.27 in the “1st case.” Increasing the number of hours of possible use of the robot (“2nd case”), the ICER has a negative value (€ −193.47). This means that the operational machines therapy is not only 2.45 times more effective than the conventional one in the case of patients recovering independence in walk (as shown by the Overall OR), but also the therapy is even more economically sustainable, as it allows to save € 193.47.

**Figure 7:**
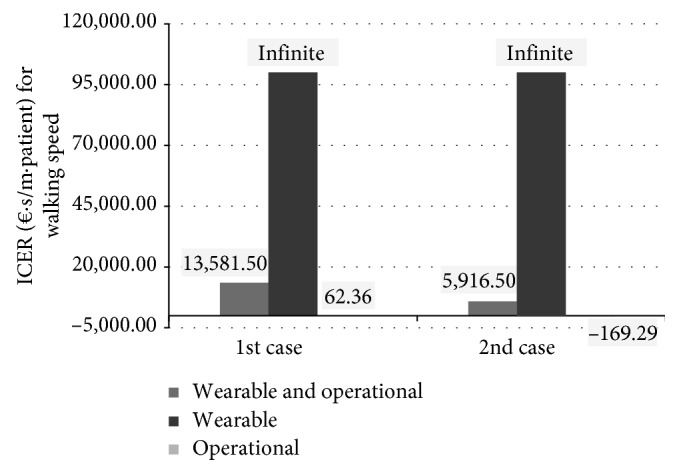
ICER values related to the independence in walking in the different cases based on the type of robot used and on the hours of potential use of the robot. Divergent ICER, for graphic purposes, set equal to € 100,000.00.

Figure 7 shows ICER values of patients recovering average walking speed. The ICER of the operational machines therapy is 62.36 (€/patient)/(m/s) in the “1st case”. This means that the robotic therapy costs approximately € 60 more than the conventional one per patient per meter per second of recovered walking speed. In the “2nd case,” the ICER is negative (−169.29 €/patient/m/s).

Results are very different for wearable robots: the ICER for them is divergent being present a zero at denominator in (8) (Overall MD = 0 m/s both in the “1st case” and in the “2nd case”).

## 4. Conclusions

Robot-mediated therapy has proven to be more effective than conventional therapy in the treatment of stroke-affected patients. Overall OR results show that the robotic therapy enables a larger number of patients to recover independence in walk, compared to the conventional therapy. This applies to all analysed cases, being the therapy based on the use of wearable robots or operational machines and being the patients in a chronic or subacute phase. This is particularly interesting since the time that patients in group A spent for robotic therapy is less than the time that patients in group B spent for conventional therapy. In the analysed studies, the duration of the rehabilitation session and the total number of sessions are the same for both groups. However, for patients in group A, about half of the session time is not spent training with the robot but rather setting-up the machine. In conventional therapy for patients in group B, the session is entirely dedicated to the rehabilitation procedure. The therapy based on the use of operational machines is the most effective treatment, with the highest Overall OR. This may be due to limitations of wearable robots. For instance, in the case of exoskeletons when the coupled joint-links are not perfectly aligned with the human joints, undesired high forces are produced [25]. This makes the robot a danger to the patient, as well as an obstacle to his/her movement. This does not happen with operational machines. Operational machines are more effective for patients in the subacute phase, whereas wearable robots are more effective for patients in the chronic phase. These results, however, are not statistically significant and require further study in the future. In terms of secondary outcome, robot-mediated therapy has the same effectiveness as conventional therapy. To sum up, robotic therapy is particularly effective in the treatment of critical patients, who are unable to walk independently before starting the rehabilitation process. For critical patients, the most important goal is to recover the walking autonomy, which is more easily achieved through robotic therapy. For patients who are already able to walk autonomously, the main goal is to recover walking speed; thus, the robotic therapy is not particularly convenient for them. Further studies are needed to assess the effectiveness of robot-mediated therapy with wearable robots to achieve the secondary outcome. Current results on this aspect are still not statistically significant.

As for therapy acceptability, patients who underwent robotic therapy with wearable robots have the lowest number of dropouts. This result is rather unexpected, as the authors thought that robot-mediated therapy would not be well accepted. A possible explanation may lie in the fact that the therapist is always present during the sessions, supervising the patient, prompting his/her active participation, and making him/her feel safe. In addition to this, the robot reduces the physical effort required to the patient and relieves his/her fatigue, especially when he/she is in difficulty. Another element to take into consideration is that the wearable robot allows patients to walk as of the very first session. This could have a positive impact on the patient's psychological response, by increasing his/her self-confidence and motivation to keep on training.

As for the economic point of view, robotic therapy based on the use of wearable robots has proven to be very expensive. Costs decrease as the hours of possible use of the robot increase. The gap between the cost of robotic and conventional therapies is considerable. Robotic therapy based on the use of operational machines is the most economically sustainable method due to the low purchasing cost. It must be said, however, that the cost of robot-mediated and conventional therapies is estimated based on the assumption that training time is the same for both therapies. The fact that the duration of the rehabilitation therapy is the same for patients in both groups A and B is based on what is reported in the articles included in this study. This, in addition to the lack of information on posttraining patients quality of life, means that the authors can evaluate only some of the direct medical costs of the therapy. It is not possible to make a comparison between the two rehabilitation approaches in terms of nonmedical direct costs (i.e., social services, home care, transportation, etc.) nor indirect costs (i.e., working days lost by the patient due to treatment and health care, working days lost in terms of lower productivity of working patients, etc.). Also, the fact that patients of both groups (A and B) underwent a programme with a similar structure does not allow to analyse other possible cost differences related, for instance, to days of hospitalisation for patients locomotion recovery. The authors, thus, highly recommend organizing clinical trials differently. Rather than having all patients undergoing a rehabilitation therapy, which has the same duration, it might be useful to set “targets” (sufficient values of effectiveness) and assess patients' recovery time against such “targets.” This would allow for a more realistic assessment of the cost of robot-mediated and conventional therapies, which takes into account rest days for patients to achieve “targets” and the other aspects mentioned above. Future clinical trials should also consider different methods for a proper assessment of nonmedical direct and indirect costs.

Taking into account both economic aspect and effectiveness, the cost difference between robotic therapy and conventional therapy is reduced. ICER results for the primary outcome show that the therapy based on wearable robots is more effective than the conventional one but also more expensive. This trend is even more evident if we consider ICER results for the secondary outcome. In this case, the ICER is divergent. This means that an infinite amount of resources would have to be spent to increase the patient's walking speed. In other words, robot-mediated therapy based on the use of wearable robots has a cost for benefit equal to 0. It is “infinitely” less efficient than conventional therapy. On the contrary, rehabilitation therapy based on operational machines is the most cost-effective one, as ICER values are very low and in some cases, even negative.

In conclusion, the study shows that robotic therapy based on the use of operational machines is the most efficient strategy. It is much more effective than the conventional one, with statistically significant results, both in terms of patients' recovery of walking ability and walking speed. It is also much more economically sustainable than robotic therapy based on the use of wearable robots, as its cost is similar, if not lower, than the cost of conventional therapy. However, the therapy which has the highest patients' acceptability is the one based on the use of wearable robots.

The investigated topic, and in general the assessment studies on medical devices, is relatively new with respect to the studies on drugs, the scientific evidence is sparse, and the attempts to collect information are challenging but the need of the assessment for medical device is crucial for supporting the decision-making process [26].

## Figures and Tables

**Table 1:** Costs analysis of robotic therapy versus conventional therapy: parameters and cost estimate results.

	Robotic		Conventional
	1st case	2nd case	
*Parameters*			
*C*_h_^therapist^ (€/h)	20.00	20.00	20.00
*n* ^therapists^	1	1	1.19
*t* ^session^ (min)	52.72	52.72	52.72
*n* ^sessions^	17.91	17.91	17.91
*C*_Robot purchase_ (€)	225,000.00	225,000.00	—
*y* ^amortization^ (years)	5	5	—
*C*_y_^maintenance^ (€/year)	22,500.00	22,500.00	—
Daily robot use (hours per day)	7.12	12	—
Weekly robot use (days per week)	5	6	—

*Results*			
*C*_h_^therapy^ (€/h)	56.46	38.03	23.80
*C*_session_^therapy^ (€/session)	52.44	35.32	22.10
*C*_total_^patient^ (€)	1,023.36	716.76	480.10

**Table 2:** Hourly cost and total cost: comparison between conventional, wearable robots, and operational machines therapies.

	Conventional	Wearable robot		Operational robot	
		1st case	2nd case	1st case	2nd case
*C*_h_^therapy^ (€/h)	23.80	73.48	46.44	24.86	22.40
*C*_total_^patient^ (€)	480.10	1,353.71	866.21	491.00	458.56
